# Effect of Diet Composition on Excreta Composition and Ammonia Emissions from Growing-Finishing Pigs

**DOI:** 10.3390/ani12030229

**Published:** 2022-01-18

**Authors:** Phung Le Dinh, Carola M. C. van der Peet-Schwering, Nico W. M. Ogink, André J. A. Aarnink

**Affiliations:** 1 Wageningen Livestock Research, Wageningen University and Research, 6708 WD Wageningen, The Netherlands; ldphung@hueuni.edu.vn (P.L.D.); gvdpeet@wxs.nl (C.M.C.v.d.P.-S.); nico.ogink@wur.nl (N.W.M.O.); 2 Faculty of Animal Sciences and Veterinary Medicine, University of Agriculture and Forestry, Hue University, 102 Phung Hung, Hue, Vietnam

**Keywords:** dietary manipulation, ammonia emission, excreta composition, growing and finishing pigs

## Abstract

**Simple Summary:**

Pig production leads to high levels of ammonia emissions, which in turn causes environmental pollution. This paper, therefore, looks at the possibilities for reducing ammonia emissions. Reducing ammonia precursors in diets and acidifying the urine and manure pH by acidifying diets make it possible to reduce the ammonia emissions from pig production facilities. The present study tested the impact of decreased crude protein or acidifying diets on urine and manure composition and ammonia emissions from growing and finishing pig houses. We found that decreasing dietary crude protein levels reduced the ammonia emissions from the floor as well as from the pig houses, whereas acidifying diets failed to reduce ammonia emissions from the floor and the pig houses. Reducing dietary crude protein is, therefore, a positive solution to reduce ammonia emissions from pig houses.

**Abstract:**

This study aimed to investigate the impact of decreased crude protein (CP) levels (by 2% units) or acidifying diets (by adding 10 g benzoic acid/kg diet in combination with replacing a part of CaCO_3_ by about 10 g Ca-formate/kg diet) on urine, feces and manure composition and ammonia emissions from growing and finishing pig houses. Yorkshire x F1(Landrace x Yorkshire) pigs (n = 576) with an initial body weight of 24.9 ± 3.4 kg were randomly allocated to four treatments of (i) a control diet with normal protein content and no acidifying components added; (ii) a diet with 2% units CP reduction; (iii) a diet with an acidifying effect on the manure; (iv) or a diet consisting of a combination of diet (ii) and (iii). Pigs were housed in four mechanically ventilated and temperature-controlled rooms. Results showed that decreasing the dietary CP levels by 2% units reduced the ammonia emission from the floor by 46% (*p* = 0.06) and from the pig house by 31% (*p* = 0.08). Decreased CP diets reduced the total N in feces and in manure and NH_4_-N in the manure, as well as the ammonia concentration at 1 cm and 10 cm above the manure surface (*p* < 0.05). However, acidifying diets failed to reduce ammonia emissions from the floor and the pig house (*p* > 0.05). Reducing dietary crude protein is, therefore, a solution to reducing ammonia emissions from pig houses.

## 1. Introduction

Ammonia is an important pollutant gas that accelerates fine particulate formation in the atmosphere and plays a crucial role in the acidification and eutrophication of ecosystems [1]. In addition, ammonia is a well-known toxic gas that can irritate the respiratory tract at concentrations exceeding 15 ppm [2]. Livestock waste accounts for 39% of global NH_3_ emissions [3]. In Europe, approximately 80% of NH_3_ production originates from livestock facilities [4]. Pig production is globally responsible for about 15% of NH_3_ emissions associated with livestock [5]. In Europe, pig production represents nearly 25% of livestock ammonia emissions [6]. Ammonia released from buildings is the main source, accounting for about 50% of pig NH_3_ [7,8]. Therefore, ammonia emission from pig production facilities should be reduced.

In livestock production, the main source of NH_3_ is the rapid hydrolyzation of urea in urine by the fecal enzyme urease, which leads to ammonium formation in an aqueous medium [9]. Another minor source of ammonia is the degradation of undigested proteins in manure. In the liquid phase, total ammoniacal N is in a state of equilibrium between ionized NH_4_^+^ and unionized NH_3_. This equilibrium is influenced by temperature and pH. Higher temperature and pH enhance NH_3_ concentration. At pH values below 7, nearly all ammoniacal N is in its ionized form. At pH above 7, the unionized fraction increases significantly.

Dietary nitrogen is the main precursor of ammonia production from pig production facilities. This ammonia emission depends on many factors, e.g., animal housing (floor type, ambient temperature and ventilation), animal (animal physiological stage, genotype/line and gender), diet (dietary crude protein, fiber type and level, feed additives such as acidifying salts and electrolyte balance), feeding management (feeding level, phase feeding), the drinking system and the manure removal system.

Feed and feeding manipulation have been shown to be effective measures for reducing ammonia emissions from pig production facilities. Reducing dietary crude protein (CP) or acidifying diets have been studied for their capacity to reduce ammonia emissions [10]. However, in most studies, ammonia emissions were measured in vitro or measured above the manure pit. The objective of this study is to study the impact of dietary measures, including reduced dietary crude protein level or acidifying salt supplementation on ammonia emissions from pig houses, urine and manure compositions.

## 2. Materials and Methods

The Animal Welfare Body of Wageningen Research, the Netherlands (IvD_WR) considered that this type of research did not fall under the legislation for the protection of animals used for scientific purposes, national decree-law 113/2013 (2010-63-EU directive). Therefore, this research got approval for ethical exemption.

### 2.1. Animals, Experimental Design, Diets and Housing

A total of 576 Yorkshire x F1 (Landrace x Yorkshire) pigs with an initial body weight of 24.9 ± 3.4 kg (mean ± standard deviation) at 63.3 ± 3.0 days old were randomly allocated into four treatments of (i) a control diet (CON) with normal protein content and no acidifying components added; (ii) a diet with a 2% reduced protein content (RDP); (iii) a diet with an acidifying effect (AD) on the manure; (iv) and a diet consisting of a combination (CD) of diet (ii) and (iii). Acidifying diets were achieved by adding benzoic acid (10 g/kg) in combination with replacing a part of the CaCO_3_ by approximately 10 g Ca-formate per 1 kg of the diet (on a Ca-equivalent basis). 

The experiment lasted for 110 days. Pigs were fed a starter diet for five weeks, a grower diet for four weeks, and a finisher diet for seven weeks. Diets were formulated to have similar contents of net energy, dietary electrolyte balance, crude fiber and non-starch polysaccharides but differed in crude protein levels, benzoic acid and Ca-formate contents (Table 1 and Table 2). All diets were supplemented with synthetic Lysine (Lys), Methionine (Met), Tryptophan (Trp) and Threonine (Thr) up to the level of the animals’ requirement [11] based on apparent ileal amino acid digestibility (Table 1). 

Pigs were fed ad libitum until they reached 70 kg live weight, after which the amount of feed provided was adjusted, gradually increasing from 2.45 to 2.65 kg until the pigs reached the weight of 120 kg. Feed was delivered once daily in a dry feeder at a single feeding place. Water was freely available via a drinking bowl. Feed and water intake were recorded every day. All pigs were weighed at the beginning and at the end of the experimental period, just before the morning feeding. In addition, at each feeding transition, five and nine weeks after the start of the experiment, pigs in four pens of each room were weighed. Average daily weight gain and feed efficiency were obtained from the feed intake and the increase in body weight (BW) during the experimental period. The daily water intake per pig per day during the 110-day experimental period was 5.1 l per day. All pigs were slaughtered at the end of the experiment. Slaughtering BW, lean meat, muscle thickness and backfat thickness were measured according to Lambooij et al. [12].

Pigs were housed in 48 pens in four similar, mechanically ventilated and temperature-controlled rooms, at 12 pens per room. Rooms were fully separated from other rooms. All pigs in one room were fed the same starter, grower and finisher diet. Each pen had an equal number of male and female pigs. The size of the pens in rooms 9 (acidifying diet treatment) and 10 (reduced crude protein diet treatment) was 2.5 × 5.0 m, with a slatted floor at the rear (2.5 × 2.0 m) and the front (2.5 × 1.15 m) and a solid, convex floor in between. The size of the pens in rooms 11 (control diet treatment) and 12 (combined diet treatment) was 2.5 × 4.85 m, with a slatted floor at the rear (2.5 × 1.5 m) and the front (2.5 × 0.55 m) and a solid, convex floor in between.

Ventilation was achieved by central fans at the end of a ventilation channel. These fans sucked the air from outside into the rooms. Each room had two exhaust ducts with a measuring fan and a valve to control the amount of air that went through the ventilator. For each measuring fan, a calibration line was made on the spot with a measuring fan that was calibrated in a wind tunnel. The number of rotations of the measuring fan was counted, and the corresponding number of pulses was logged in the climate control system. Temperatures and humidities of the rooms were measured near the exhaust air ducts and were recorded every minute. The average temperatures and humidities of the rooms during the 110-day experimental period were 22.1 °C and 56.7%; 23.6 °C and 67.0%; 23.2 °C and 60.1%; 23.0 °C and 58.6% for the rooms with control, reduced CP, manure acidifying and combined diets, respectively.

Experimental diets were analyzed for dry matter, ash, crude protein, minerals, crude fiber, fat, starch, sugar, gross energy, some essential amino acids (AA), benzoic and formic acids. The AA (except Met, Cys and Trp) were assayed by ion-exchange column chromatography after hydrolysis for 23 h in HCl (6 mol L^−1^). Cystine and Met were determined as cysteic acid and methionine sulfone after oxidation with performic acid before hydrolysis [13]. Tryptophane was determined according to Sato et al. [14]. Benzoic acid was analyzed with liquid chromatography with UV detection after aqueous extraction and purification of the samples. Regarding formic acid, after hydrolysis, an analysis of the extracts was performed using ion chromatography with conductivity detection. Benzoic acid and formic acid were analyzed at the lab of NutriControl, Veghel in the Netherlands.

Starch content was determined enzymatically according to the amyloglucosidase/hexokinase method (NEN 3574). Sugar was assayed according to the non-starch polysaccharides (NSP) procedure [15]. Crude fiber was determined gravimetrically after treatment with sulfuric acid and potassium hydroxide (ISO/DIS 6895). For total fat, samples were hydrolyzed with chloric acid, followed by extraction of fat with petroleum ether. The Ca, P, Mg, Na, K, Cu and Zn contents were determined using inductively coupled plasma atomic emission spectrometry (ICP-AES). The Cl content was determined by the potentiometric titration of water-diluted solid samples with the chloride specific ion electrode (Jenway Chloride Meter, model PCLM3, Staffordshire, UK). For sulfate, samples were extracted with chloric acid. Sulfate was separated with ion chromatography using a water // sodium hydroxide gradient and an Ionpac AS 11 (Dionex, ThermoFisher Scientific, Waltham, MA, USA) as the column. Detection takes place by suppressed electric conductivity. Identification and quantification were achieved using an external standard solution. The DM was determined gravimetrically after 4 h at 103 °C (ISO 6496). The content of ash was determined gravimetrically after ashing at 550 °C (ISO 5984). Nitrogen content was determined by the Kjeldahl method (ISO 5983). The analyzed dietary CP levels of the starter diets were 18.5% for CON and AD treatments and 16.5% for RDP and CD treatments. The analyzed dietary CP levels of the grower diets were 16.03% for CON and AD treatments and 14.02% for RDP and CD treatments. The analyzed dietary CP levels of the finisher diets were 15.52% for CON and AD treatments and 13.52% for RDP and CD treatments (Table 2).

### 2.2. Sample Collection, Measurements and Calculations

Measurements of ammonia emissions, fresh urine and feces, urine patches and manure characteristics were done every three weeks. In total, there were four rounds of measurements. In each round, the measurement lasted for four days. First, the ammonia emissions from each of the four pig rooms were continuously measured for one day. On the next day, fresh urine, feces, urine patches and manure samples from the manure pit were collected. On the third day, the manure pit was cleaned and filled with water up to 10 cm below the slatted floor for measuring ammonia emissions from the floor on the fourth day. 

#### 2.2.1. Ammonia Emissions from the Animal House

On the measurement days, as mentioned in paragraph 2.2, in each room, continuous measurements of ammonia concentration, ventilation flow, temperature and relative humidity were conducted. Ammonia concentrations were measured with a NOx (Model 200A Chemiluminescent NOx Analyzer, Teledyne API, San Diego, CA, USA) by sampling the inlet and outlet air inside the shafts of the fans and after ammonia had been converted to NO at 775 °C, as described by Ogink et al. [16]. The analyzer was calibrated every three weeks. The ventilation rate was measured with a calibrated anemometer installed in the exhaust chimney in each room, as described by Mosquera et al. [17]. One anemometer was used in each room. Temperature and humidity of the outgoing air were measured just under the opening of the shaft by an Escort T/RH sensor in combination with a logging system (ESCORT iLog Humidity Loggers, Aesch, Switzerland). Ammonia emissions from the different rooms were calculated from the difference in ammonia concentrations of the outgoing air and incoming air and from the ventilation rate. This included the ammonia emissions from the manure pit and the floor.

#### 2.2.2. Ammonia Emissions from Floor

After the removal of manure from the manure pit, the manure pit was filled with water up to 10 cm below the slatted floor. This was done to make ammonia emissions from the manure pit equal to 0. The ammonia concentration, temperature, humidity and ventilation rate were measured on the next day. Ammonia emissions from the floor were measured and calculated as they were with ammonia emissions from the animal house.

#### 2.2.3. Fresh Urine and Feces, Urine Patches and Manure Characteristics

Fresh urine and fresh feces characteristics: Single urine and feces samples were taken from 12 pigs (6 male and 6 female, randomly chosen from 4 pens) per dietary treatment. The samples from each pig were kept separately in the first instance. Approximately 2 mL of urine from each urine sample was inserted with a pipette in a jar with 2 mL acid HCl 1 M to stop urea conversion. Therefore, there were 2 mL of acid and 24 mL of urine in the jar. In the remaining urine, the pH was determined on the spot. In the lab, the fresh urine samples without acid were joined together on an equal mass basis and analyzed for NH_4_-N, Total N and potassium. In these samples, the urine-urea before NH_4_-N analysis was first converted by the addition of urease. The fresh urine samples in acid were analyzed for NH_4_-N. 

The fresh feces samples were also joined together on an equal mass basis and analyzed for NH_4_-N, N-total and potassium. Ammonium-N was determined spectrophotometrically according to NEN 6472 [18]. Manure pH was measured by a pH meter (Orion 520A) with an electrode (Orion 9104 SC). Potassium content was determined using an inductively coupled plasma atomic emission spectrometer (ICP-AES). Total nitrogen content was determined by the Kjeldahl method (ISO 5983).

Urine patch characteristics: The ammonium content and pH of urine were determined through the “filter method”. For this purpose, 10 samples were taken at 10 representative locations in two pens of each room. Representative means of samples were taken from locations that were very soiled. Samples were taken by siphoning the urine through glass fiber filters, two of which were placed at each sample location. This included one filter in a jar with 10 mL acid HCl 1 M and the other filter in a jar with 10 mL demineralized water. 

Ten filters were combined into one composite sample in the jar with acid and one composite sample in the jar with water. After mixing, the pH was measured on the spot in the jar of filters in water. In the chemical laboratory, the jars with filters in water were analyzed for NH_4_-N and potassium content. It was ensured that all the urea was converted to ammonium (addition urease). Potassium was analyzed to determine how the NH_4_-N/K ratio changed from fresh to urine patches. This provides an indication of the loss of NH_4_-N from the urine patch. The level of potassium in the urine puddles relative to the potassium content in the fresh urine gives an indication of the water evaporation. The jars with acid filters were analyzed for NH_4_-N. 

Upper layer manure characteristics: Single aggregate samples of the uppermost layer of manure in the manure pit were collected via the ‘filter’ method (absorption of samples of the uppermost layer of manure). We took 10 samples that were evenly distributed over the manure pit of two pens/room. At each sample site, two filters were taken. This included one filter in a jar with 10 mL acid HCl 1 M and the other filter in a jar with 10 mL demineralized water. Samples of 10 locations were combined into one composite sample in the container with acid and one composite sample in the jar with water. After mixing, the pH was measured on the spot in the jars with water. In the chemical laboratory, in the jar with water, the ammonium and potassium contents were analyzed. 

Manure characteristics: We took a one-liter sample of the manure from the manure pit during the discharge of the manure into the interim storage before it was pumped into an outside storage. During the discharge, a sample was taken at regular moments from the manure stream. The pH of the sample was measured directly in the manure bottle. At the lab, DM, ash, NH_4_-N, total N, potassium, sodium, chloride, phosphor, calcium and total volatile fatty acids concentrations were determined. In addition, from each bucket, two samples of 10 g were taken in fully airtight sealed jars of 60 mL with a rubber septum, which had been pre-weighed. The samples in airtight jars were used for determining the total inorganic carbonate (TIC) and alkalinity/acidity. All samples were stored at −20 °C until the analyses. Volatile fatty acids in the manure were measured using a Packard 427 gas chromatograph, equipped with a flame ionization detector. TIC was measured by acidifying manure samples that had been stored in gas-tight bottles and measuring the volume of CO_2_ evolved [19]. The buffer capacity was determined by measuring the pH change in response to the addition of a small quantity of strong acid (HCl) or strong base (NaOH). Other manure characteristics were analyzed according to protocols, as mentioned in feed analyses.

#### 2.2.4. Ammonia Concentration at 1 cm and 10 cm above the Manure Pit

The levels of ammonia concentration at 1 cm and 10 cm above the manure pit were measured once every three weeks. Each time the measurement lasted for about 30 min. We measured it with a NOx monitor (Teledyne Model T200, San Diego, CA, USA), as described by Ogink et al. [16], every minute using a data logger system (Campbell Scientific, Inc, Logan, UT, USA). The final recorded ammonia concentration was the average of all one-minute recorded values.

#### 2.2.5. pH and Temperature Gradient in Manure

The pH and temperature gradient in the manure of two pens/room were measured. The pH and temperature were measured at 0.50 and 5 cm from the surface of the manure. 

### 2.3. Statistical Analysis

The effects of reduced crude protein or manure acidifying diets on animal performance (daily weight gain, feed intake, feed conversion ratio, slaughter data) were analyzed on pen level using ANOVA GenStat (Genstat Committee, 2014, VSN International Ltd, Hemel Hempstead, UK) with the following model.

Y_ij_ = μ + T_i_ + e_ij_ (1)

where: Y_ij_: dependent variables; μ: overall mean; T_i_ = effects of diet (i = 1 CON; i = 2 = RCP; i = 3 = AD; i = 4 = CD); e_ij_ = residual error.

The effects of reduced crude protein or manure acidifying diets on ammonia emission, urine, feces and manure characteristics were analyzed at room level using ANOVA GenStat (Genstat Committee, 2014, VSN International Ltd, Hemel Hempstead, UK) with the following model.

Y_jklm_ = μ + CP_i_ + AD_j_ + BR_K_ + BW_1_ + e_ijklm_ (2)

where: Y_ijklm_: dependent variables, μ: overall mean; CP_i_= effects of protein (i = 1= normal diet; i = 2 = reduced CP diet); AD_j_= effects of acidifying diet (j = 1 = normal diet; j = 2 = acidifying diet); BR_k_ = effects of room block (k = 1 = room 11 and 12, k = 2 = room 9 and 10); BW_l_ = Effect of sample collection day block (l = 4 = day of sample collection, once every three weeks; four times); e_ijklm_: residual error.

The interaction effect between the reduced protein and acidifying diet treatments could not be included in the model, as it is intertwined with the effect of the room block. Research by Bakker et al. [20] has shown that the effects of feed measures aimed at reducing ammonia emissions by reducing the protein content and lowering the pH of urine and manure are additive and therefore do not interact with each other.

Data are presented as an arithmetic mean. A natural log transformation was applied to ammonia emissions since they were not normally distributed.

## 3. Results

### 3.1. Effects of Experimental Diets on Daily Gain, Daily Feed Intake and Feed Efficiency

Effects of experimental diets on final body weight (BW), average daily feed intake (ADFI), average daily weight gain (ADG) and feed conversion ratio (FCR) are presented in Table 3. There were no significant differences in final BW among treatments. Average daily weight gain and ADFI were lowest in the CON treatment, followed by CD and AD treatments, and highest in the RCP treatment (*p* < 0.001). FCR did not show any significant difference among the treatments (*p* > 0.05).

### 3.2. Effects of Experimental Diets on Slaughter Quality

Effects of experimental diets on meat performance are presented in Table 4. Results show that experimental diets did not affect slaughtering BW, lean meat percentage or backfat thickness (*p* > 0.05). However, muscle thickness was highest in CD and lowest in RCP diets (*p* = 0.001). 

### 3.3. Effects of Experimental Diets on Urine and Feces Characteristics

Characteristics of urine and feces among the experimental pigs are presented in Table 5. The total N in feces was reduced when pigs were fed reduced crude protein diets (*p* = 0.01). Acidifying diets reduced pH fresh urine at the spot measured directly inside the pig house (*p* < 0.001) and pH urine soiled (*p* = 0.07) and NH_4_-N of upper manure in the jar with water (*p* = 0.06). Other items did not show significant differences (*p* > 0.05).

### 3.4. Effects of Experimental Diets on Manure Characteristics

Manure characteristics among the experimental pigs are presented in Table 6. Reducing dietary crude protein level or acidifying diets did not affect manure DM, concentrations of total ash, K, Na, P, Cl, Ca, total volatile fatty acid (VFA) and acetic, propionic, iso-butyric, iso-pentanoic, pentanoic acids (*p* > 0.05). Manure pH measured at the spot was lower with reduced CP diets (*p* = 0.06), while acidifying diets did not affect manure pH measured on the spot (*p* > 0.05). Total N was reduced when pigs were fed reduced CP diets (*p* = 0.04) or acidifying diets (*p* = 0.07). Reducing dietary CP levels or acidifying diets reduced NH_4_-N in the manure (*p* < 0.05). The buffer capacity was significantly increased when CP was reduced (*p* < 0.001). Manure TIC was lower when pigs were fed reduced CP or acidifying diets (*p* < 0.001).

### 3.5. Effects of Experimental Diets on Ammonia Emission

Table 7 shows the effects of experimental diets on pH in manure at depths of 0.5 cm and 5 cm from the manure surface, ammonia concentration at 1 cm and 10 cm above the manure surface and ammonia emissions from the floor and the animal house. Reducing dietary CP level reduced the manure pH measured to a depth of 0.5 cm (*p* = 0.07) and 5 cm (*p* = 0.003) from the manure surface; ammonia concentration at 1 cm (*p* = 0.02) and at 10 cm (*p* = 0.02) above the manure surface; and ammonia emission from the floor (*p* =0.06) and from the animal house (*p* = 0.08). However, acidifying diets did not affect those parameters (*p* > 0.05).

## 4. Discussion

Dietary nitrogen is the main cause of high levels of ammonia production on pig farms. According to Aarnink [21], 30% of the nitrogen in the diet is deposited in the body of growing-finishing pigs and 70% is excreted via urine and feces. Almost half of the excreted nitrogen is emitted during the storage of the manure inside the pig house and during the surface application of the manure. Changing the pig feed and the feeding strategy has been shown to be effective strategies to reduce ammonia emissions in the pig production facilities [10]. Two main feed and feeding strategies to reduce emissions are: (i) reducing nitrogen excretion by lowering the protein level in the diet and (ii) lowering the pH of the manure (by lowering the pH of feces and/or lowering the pH of urine). The latter can be achieved by changing the electrolyte balance of the diet. However, these two strategies were mainly tested in vitro or in controlled conditions such as restricted water intake [22,23,24,25,26] or measured above the manure pits [27,28]. In our current study, we applied CP levels (from 18.5% to 16.5%; 16.5% to 14.5%; and 15.5% to 13.5% for starter, grower and finisher diets, respectively) close to the target levels for the CP content in commercial diets described in the Framework code as good agricultural practice for reducing ammonia emissions (as published by the UN economic commission in Europe) [29]. Furthermore, the fact that we measured ammonia emissions from the floor and the pig house gave the results of our studies realistic meaning and produced ramifications that can be applied directly in commercial pig farms.

In our study, reduced CP diets decreased total N in feces (*p* = 0.01) and in manure (*p* = 0.04), urinary NH_4_-N in jar with acid (*p* = 0.09), NH_4_-N of urine patches in jar with acid (*p* = 0.09), manure NH_4_-N (*p* = 0.02), manure TIC (*p* = 0.009) and increased manure buffer capacity (*p* = 0.001). Reduced CP diets have been shown in the literature to decrease N excretion in feces, urine and manure (urine + feces). The urinary nitrogen of finishing pigs decreased linearly by lowering dietary CP levels from 16.8 to 15.9; 14.8 and 13.6% [30]. According to O’Connell et al. [31], finishing pigs that were offered diets containing 14% CP excreted less nitrogen, urinary nitrogen and fecal nitrogen than those offered diets containing 16% CP. Reduced CP diets supplemented with crystalline amino acids (AA) have been shown to reduce fecal nitrogen excretions by 25–30% [32,33]. According to Portejoie et al. [34], lowering dietary CP from 20% to 16% and 12%, while maintaining similar levels of essential AAs, reduced total N content in manure from 5.48 to 4.30 and 3.05 g N kg^−1^, respectively. 

The pH has a direct effect on ammonia emissions, since both urinary nitrogen and fecal nitrogen are further converted to ammonium (NH_4_^+^) at a low pH and to ammonia (NH_3_) at a high pH [35]. In our study, reduced CP diets reduced the pH at depths of both 0.5 cm (*p* = 0.07) and 5 cm (*p* = 0.003) from the manure surface and decreased manure pH on the spot (*p* = 0.06) and pH of urine soiled on the floor (*p =* 0.07); this confirms previous findings. According to Portejoie et al. [34], the pH of the manure decreased by 1.3 units when dietary CP decreased from 20% to 12%. According to Shriver et al. [36] and Aarnink and Verstegen [35], reduced CP diets supplemented with AA decrease not only nitrogen excretion but also manure pH, which inhibits the conversion of ammonium (NH_4_^+^) to ammonia, thus reducing ammonia emissions.

Ammonia emissions depend on urine, feces and manure characteristics, such as total N, NH_4_-N and pH. Reduced CP diets decreased manure pH and N excretions in feces and manure, and thus, the ammonia concentrations and emissions. In our study, a 2% unit reduction in dietary CP reduced ammonia concentrations at 1 cm and 10 cm above the manure surface by 49% and 24%, respectively; ammonia emission from the floor and from the animal house were reduced by 46% and 31%, respectively. Madrid et al. [37] found that a reduction in dietary CP of 1% and 2% of growing and finishing pigs, accompanied by supplementation with crystalline amino acid, reduced ammonia emissions from the chambers (pigs were kept in chambers) by 19.9% and 27.3%, respectively. According to Hansen et al. [38], ammonia emissions from pig houses were reduced by 23% when finishing pigs were fed a low-protein, compared to a standard protein, diet containing 136 or 159 g crude protein/kg diet, respectively (2.3% unit reduction). It is well documented in the literature that for each 1% unit reduction in dietary CP, when combined with AA supplementation, the estimated ammonia emissions (measured via in vitro studies or measured directly above the manure pit) are reduced by 10% [24,39,40]. The total reduction of ammonia emissions from the animal houses, by lowering dietary CP levels in the current study and others, was higher than that from in vitro studies or from the manure pit, which is in line with our study finding a stronger effect of CP reduction on floor emissions compared to the animal house emissions.

Acidifying diets to lower the urine and manure pH is one of the feed strategies to reduce ammonia emissions. In our study, acidifying diets by adding benzoic acid (10 g/kg) combined with replacing a portion of the CaCO_3_ by about 10 g Ca-formate/kg diet (on Ca–equivalent basis) reduced the pH of fresh urine on the spot (*p* < 0.01) and the pH of soiled urine (*p* = 0.07), but did not affect the manure pH measured on the spot, nor the pH at depths of 0.5 cm and 5 cm from the manure surface (*p* > 0.05). According to Bühler et al. [41], the addition of benzoic acid (1%) to the diet of pigs from 26 kg to 100 kg reduced urinary pH by about one pH-unit. Kluge et al. [42] reported that the relationship between benzoic acid intake and the urinary pH response was linear. Marie-Line Daumer et al. [43] declared that nitrogen excretion and the urine and manure pH of growing pigs were decreased when 1% benzoic acid was added to the diet. Some kinds of acid salts have been added to diets to reduce ammonia emissions based on the principle of reducing the pH. According to Kim et al. [44], urine pH was reduced when replacing di-calcium-phosphate (CaHPO_4_) and calcium carbonate (CaCO_3_) with phosphoric acid (H_3_PO_4_) and calcium sulfate (CaSO_4_). Canh et al. [23] reported that replacing calcium carbonate with calcium sulfate, calcium chloride (CaCl_2_) or calcium benzoate (Ca(C_7_H_5_O_2_)_2_) significantly lowered the pH of urine and manure.

Acidifying diets did not reduce manure pH or ammonia concentrations above the manure surface, and thus, ammonia emissions from the pig house (*p* > 0.05). While supplementing with acid salts and/or organic acids has been well proven in in vitro studies [23,43,44] to reduce urinary and manure pH, and thus ammonia emissions, it was not confirmed in our in vivo studies. This is in opposition to the findings of Hansen et al. [45], where adding 3% of benzoic acid to the diet of finishing pigs reduced ammonia emissions from the chamber by 57%. Supplementing diets with a mixture of acid salt (70% benzoic acid, 16.5% calcium salts, 6.5% formic acid and 7.0% propionic acid) comprising 1% and 2% of the feed, respectively, for starter and growing-finishing pigs reduced ammonia emission from the pig houses by 40% [46]. Probably, different amounts of acid salts and organic acids used in the various studies accounted for inconsistent results in terms of ammonia emission reduction from the pig house. In addition, in our study, manure has a high buffer capacity, and that might also be the reason that the effect of the acidifying diet was rather low. The buffer capacity of the protein treatment was the highest. This could point to an interaction between the protein and acidification treatment in the CD treatment. In this study, reduced protein and acidifying diet treatments could not be tested, as they were intertwined with the effect of room block. If an interaction effect of both treatments occurred, the single effect of acidification could be underestimated.

Reducing CP levels can decrease ammonia emissions from the animal house. Therefore, this dietary measure can be applied in growing and finishing pig production to reduce ammonia emissions. One of the important concerns of the producers is whether animal performance will be reduced when reducing CP levels are applied to reduce ammonia emissions. The current study found that reducing CP increased ADG (*p* < 0.05). This was the result of increasing the daily feed intake (*p* < 0.05). The feed conversion ratio was not affected by reduced CP diets (*p* > 0.05). Reduced CP diets did not affect meat performance (slaughtering body weight, lean meat, backfat thickness), except that the muscle thickness was reduced when lowering CP and acidifying diets. These results are in line with previous findings. Madrid et al. [37] found that a 2% reduction in the dietary CP of growing and finishing pigs supplemented with crystalline amino acid had no detrimental effects on growth performance, but did reduce muscle depth at the time of slaughter. Similarly, a 2.3% reduction of dietary CP for finishing pigs (from 15.9% to 13.6%) while simultaneously supplementing the indispensable amino acids did not impair growth, feed utilization, nor meat percentage [38]. Nørgaard et al. [30] indicated that the CP could be decreased to 136 g/kg as-fed for growing pigs in the weight range of 50–100 kg with no negative impact on growth performance and carcass traits. Le et al. [28] and Le et al. [27] found similar ADGs of pigs fed diets with CP levels of 12%, 15% and 18% supplemented with essential amino acids. Oldenburg and Heinrichs [47] found no negative effects on the performance and leanness of pigs between 50 and 110 kg when CP levels in diets were reduced from 17% to 13.5%. According to Lopez et al. [48] and Hahn et al. [49], pigs fed reduced-CP diets (a reduction of 3.5 to 4%) supplemented with AA had similar carcass characteristics to pigs fed diets with a normal CP.

## 5. Conclusions

This study demonstrated that decreasing the dietary crude protein level by 2% units reduced ammonia emission from the floor and from the pig house by 46% and 31%, respectively. Reduced CP diets decreased the total N in feces and in manure, manure NH_4_-N (*p* < 0.05), pH at depths of 0.5 cm (*p* = 0.07) and at 5 cm (*p* = 0.003) from the manure surface, manure pH at the spot (*p* = 0.06) and pH of urine soiled on the floor (*p =* 0.07). Acidifying diets (by adding 10 g benzoic acid/kg diet as well as replacing a portion of the CaCO_3_ by about 10 g Ca-formate/kg diet) did not significantly reduce ammonia emissions from the floor and the pig house, manure pH measured at the spot, nor pH at the depths of 0.5 cm and 5 cm from the manure surface (*p* > 0.05), although the pH of fresh urine at the spot (*p* < 0.01) and the pH of soiled urine (*p* = 0.07) was reduced. The decreased crude protein diet gave a higher growth rate in growing-finishing pigs when compared with the control diet; the growth rate of the acidifying diet was in between. Reducing dietary crude protein is a solution to reducing ammonia emissions from pig houses.

## Figures and Tables

**Table 1:** Ingredient composition of experimental diets, as-fed basis.

Composition (%)	Starter Diet				Grower Diet				Finisher Diet			
	CON	RCP	AD	CD	CON	RCP	AD	CD	CON	RCP	AD	CD
Maize	11.32	12.81	11.79	12.81	5.40	5.00	5.00	5.00	5.00	5.00	5.40	5.00
Barley	24.63	24.63	24.63	24.63	19.80	22.70	19.80	22.00	19.80	19.80	19.80	19.80
Wheat	17.50	16.75	14.78	16.75	27.45	26.80	25.07	24.80	27.65	29.64	24.56	28.74
Maize feed meal	5.00	4.81	5.00	4.41	4.50	4.95	4.75	4.95	4.50	4.95	4.50	4.95
Wheat middlings		3.39	1.01	4.21	7.00	9.90	7.00	9.90	7.00	10.00	7.00	10.00
Biscuits, ground	4.93	4.38	4.93	3.94	4.30	4.00	5.00	5.00	3.50	2.80	4.50	3.00
Wheat gluten feed meal	7.30	6.90	7.22	6.90	5.00	5.00	5.00	5.00	4.50	4.50	4.50	4.90
Vegetable oil	1.61	1.92	1.82	1.90	1.50	1.60	1.95	2.00	1.00	1.00	1.40	1.35
Potato protein		0.28										
Sugar beet pulp					1.10	1.30	1.10	1.30	1.10	1.50	1.10	1.20
Soybean meal HIPRO	17.29	14.87	17.77	16.04	10.10	7.90	11.00	8.30	8.50	5.00	9.00	5.00
Soybean hulls		1.04		0.77	1.30	2.00	1.30	2.00	1.00	1.00	1.00	1.00
Rapeseed meal, fat extract	4.93		4.93		5.00		5.00		4.80	1.00	5.00	1.10
Sunflower seed meal, fat extract	1.48	0.99	1.21		4.00	2.90	3.20	3.00	3.50	3.50	3.50	3.60
Palm kernel flakes									4.40	4.90	3.80	5.00
Whey, concentrated	0.40	3.00	0.10	2.07	1.10	3.00	1.10	2.80	1.70	3.00	1.70	1.70
Limestone	1.14	1.11	0.30	0.30	0.89	0.89			0.93	0.96	0.13	0.18
Salt	0.28	0.20	0.29	0.21	0.20	0.22	0.20	0.12	0.19	0.09	0.19	0.08
Vitamin extra					0.19	0.19	0.19	0.19				
Premix ^1^	0.25	0.25	0.25	0.25	0.26	0.26	0.26	0.26	0.25	0.25	0.25	0.25
DL-Methionine	0.09	0.17	0.10	0.21	0.03	0.08	0.03	0.08		0.03		
l-Lysine HCL	0.52	0.71	0.50	0.70	0.41	0.61	0.38	0.60	0.29	0.51	0.28	0.51
l-Threonine	0.14	0.22	0.14	0.22	0.09	0.17	0.09	0.17	0.04	0.12	0.04	0.12
l-Tryptophan	0.05	0.17	0.05	0.16		0.09		0.08				
Valine		0.29		0.30								
Benzoic acid			1.00	1.00			1.00	1.00			1.00	1.00
Calcium formate			1.04	1.03			1.00	1.00			1.00	1.00

CON = control diet, RDP = reduced crude protein diet, AD = acidifying diet, CD = combined diet = reduced crude protein + acidifying diet. ^1^ The vitamin–mineral premix supplied per kg feed included 7000 IU vitamin A, 1700 IU vitamin D3, 20 IU vitamin E, 1.5 mg vitamin K, 1.5 mg vitamin B1, 4 mg vitamin B2, 11 mg d-pantothenic acid, 18 mg niacine, 18 µg vitamin B12, 0.1 mg folium acid, 1.0 mg vitamin B6, 100 mg choline chloride, 75 mg Fe, 10 mg Cu, 65 mg Zn, 30 mg Mn, 0.15 mg Co, 0.75 mg I, 0.30 mg Se.

**Table 2:** Nutrient compositions of experimental diets, as fed basis.

		Starter Diet				Grower Diet				Finisher Diet			
Composition	Unit	CON	RCP	AD	CD	CON	RCP	AD	CD	CON	RCP	AD	CD
Calculated composition													
Crude protein	%	18.50	16.50	18.50	16.50	16.03	14.00	16.03	14.02	15.52	13.52	15.52	13.50
Crude fat	%	4.37	4.63	4.59	4.57	4.21	4.24	4.70	4.71	3.86	3.82	4.29	4.20
Crude fiber	%	4.20	4.04	4.20	3.84	5.85	5.48	5.64	5.49	6.21	6.05	6.13	6.08
Crude ash	%	5.00	4.95	4.99	4.92	4.81	4.75	4.70	4.54	4.69	4.50	4.68	4.44
Moisture	%	10.67	11.69	10.44	11.31	11.53	12.27	11.28	11.94	11.68	12.23	11.42	11.52
Starch	%	39.48	40.12	38.40	39.93	39.01	40.46	37.89	39.32	38.33	39.94	37.17	39.55
Sugars	%	5.33	5.55	5.27	5.31	5.00	5.08	5.09	5.12	4.89	4.77	5.01	4.37
Non-starch polysaccharides	%	16.42	16.13	16.66	16.08	19.54	19.06	19.48	19.24	21.16	21.03	21.06	21.20
Digestible non-starch polysaccharides	%	9.08	8.80	9.10	8.70	10.30	9.92	10.29	9.92	11.51	11.17	11.32	11.05
Net energy	Kcal	2396	2396	2396	2396	2299	2299	2304	2302	2253	2255	2253	2253
Apparent ileal digestible amino acids													
Lys	%	1.02	1.02	1.02	1.02	0.79	0.79	0.79	0.79	0.68	0.68	0.68	0.68
Met	%	0.34	0.39	0.35	0.42	0.25	0.26	0.25	0.26	0.21	0.21	0.21	0.21
Met + Cys	%	0.60	0.61	0.60	0.64	0.48	0.46	0.48	0.46	0.44	0.41	0.44	0.41
Thr	%	0.66	0.66	0.66	0.66	0.51	0.51	0.52	0.52	0.44	0.44	0.45	0.44
Tryp	%	0.19	0.19	0.19	0.19	0.15	0.15	0.15	0.15	0.14	0.13	0.14	0.13
Iso-Val	%	0.61	0.53	0.61	0.53	0.50	0.41	0.50	0.42	0.47	0.38	0.47	0.38
Val	%	0.70	0.64	0.70	0.64	0.59	0.50	0.59	0.50	0.56	0.47	0.57	0.47
Calcium	%	0.70	0.70	0.70	0.70	0.65	0.65	0.62	0.62	0.62	0.61	0.62	0.61
Phosphorus total	%	0.43	0.41	0.43	0.40	0.47	0.44	0.47	0.44	0.47	0.45	0.47	0.45
Potassium	%	0.85	0.86	0.85	0.84	0.80	0.78	0.80	0.78	0.78	0.74	0.79	0.74
Sodium	%	0.17	0.17	0.17	0.17	0.14	0.18	0.14	0.14	0.14	0.14	0.14	0.14
Digestible phosphorus	%	0.27	0.28	0.27	0.27	0.27	0.27	0.27	0.27	0.25	0.26	0.26	0.25
Sulfur total	%	0.27	0.28	0.27	0.28	0.24	0.23	0.23	0.23	0.22	0.21	0.22	0.21
Dietary electrolyte balance	meq	215	220	215	220	198	200	199	201	193	195	194	195
Vit A -added	IE/kg	10,000	10,000	10,000	10,000	9719	9719	9719	9719	7758	7758	7758	7758
Vit D_3_ -added	IE/kg	2000	2000	2000	2000	1996	1996	1996	1996	1842	1842	1842	1842
Vit E -added	mg/kg	120	120	120	120	101	101	101	101	99	99	99	99
Phytase-equivalent	FU/kg	1000	1000	1000	1000	758	758	758	758	657	657	657	657
Analyzed composition													
Crude protein	%	18.6	16.2	17.9	16.8	16.2	15.1	15.7	14.4	15.6	13.7	15.6	13.6
Crude fat	%	5.0	4.9	5.3	5.4	4.4	4.3	4.4	4.7	4.1	4.1	4.4	4.4
Crude fiber	%	4.2	4.2	4.3	3.9	5.5	5.2	5.2	5.7	5.7	5.7	5.8	6.2
Crude ash	%	5.1	5.7	5.0	4.9	4.8	4.9	4.6	4.7	4.6	4.4	4.6	3.9
Sugar	%	5.4	4.9	5.2	6.0	5.1	5.1	5.6	5.0	5.1	4.4	5.0	4.4
Starch	%	35.7	36.8	35.9	36.2	36.8	38.7	38.2	36.4	37.5	40.0	37.2	37.5
Calcium	%	0.84	1.17	0.76	0.77	0.74	0.72	0.70	0.79	0.68	0.64	0.68	0.76
Potassium	%	0.81	0.83	0.84	0.84	0.79	0.75	0.76	0.76	0.76	0.72	0.78	0.81
Sodium	%	0.15	0.15	0.17	0.17	0.14	0.15	0.13	0.13	0.13	0.13	0.14	0.13
Chlorine	%	0.25	0.25	0.27	0.28	0.23	0.27	0.23	0.22	0.23	0.21	0.24	0.16
Sulfur total	%	0.22	0.26	0.24	0.26	0.19	0.23	0.17	0.23	0.17	0.22	0.17	0.22
Benzoic	%			0.87	0.65			0.70	0.88			0.83	0.94
Formic acid	%			0.53	0.64			0.48	0.60			0.58	0.62
Dietary electrolyte balance	meq	202	207	212	210	198	181	186	189	186	182	193	218

CON = control diet, RDP = reduced crude protein diet, AD = acidifying diet, CD = combined diet = reduced crude protein + acidifying diet, Dietary electrolyte balance was determined as mEq = Na + K − Cl.

**Table 3:** Effects of experimental diets on average daily feed intake, average daily weight gain and feed conversion ratio (mean ± standard deviation, *n* = 12).

Items	CON	RCP	AD	CD	*p*-Value
Initial BW (kg)	24.7 ± 2.59	25.2 ± 3.80	25.3 ± 3.84	24.7 ± 2.58	0.95
Final BW (kg)	123.8 ± 2.88	125.3 ± 3.67	123.0 ± 4.17	124.9 ± 2.78	0.33
Average daily weight gain (g/day)	876 ^a^ ± 18	922 ^b^ ± 14	898 ^c^ ± 29	894 ^c^ ± 19	<0.001
Feed intake (kg/day)	2.16 ^a^ ± 0.04	2.30 ^b^ ± 0.05	2.25 ^c^ ± 0.08	2.24 ^c^ ± 0.03	<0.001
Feed conversion ratio (kg feed/kg weight gain)	2.47 ± 0.03	2.50 ± 0.06	2.50 ± 0.10	2.50 ± 0.05	0.46

CON = control diet, RCP = reduced crude protein diet, AD = acidifying diet, CD = combined diet. ^a, b, c^ Means within rows missing a common superscript letter are different at *p <* 0.05.

**Table 4:** Effects experimental diets on meat performance (mean ± standard deviation, *n* = 12).

Items	CON	RCP	AD	CD	*p*-Value
Slaughtering BW (kg)	93.6 ± 1.8	96.1 ± 3.0	95.1 ± 3.1	96.0 ± 2.5	0.10
Lean meat (%)	59.3 ± 0.5	58.9 ± 0.5	59.1 ± 0.6	59.0 ± 0.4	0.34
Muscle thickness (mm)	65.0 ^ab^ ± 2.0	63.2 ^c^ ± 1.6	63.5 ^ac^ ± 2.6	66.5 ^b^ ± 1.8	0.001
Back fat thickness (mm)	13.7 ± 0.72	14.2 ± 0.8	14.0 ± 0.9	14.1 ± 0.6	0.34

CON = control diet, RCP = reduced crude protein diet, AD = acidifying diet, CD = combined diet. ^a, b, c^ Means within rows missing a common superscript letter are different at *p <* 0.05.

**Table 5:** Effects of experimental diets on urine and feces characteristics (mean ± standard deviation, *n* = 4).

Items	CON	RCP	AD	CD	*p*-Value	
					Protein	Acidifying
Total N in feces (g/kg)	9.23 ± 1.10	8.84 ± 0.98	9.55 ± 0.77	8.21 ± 1.06	0.01	0.61
NH_4_-N in feces (g/kg)	1.02 ± 0.18	0.95 ± 0.19	0.96 ± 0.05	0.99 ± 0.09	0.79	0.90
K in feces (g/kg)	3.34 ± 0.28	3.10 ± 0.18	3.12 ± 0.29	2.98 ± 0.09	0.11	0.15
pH fresh urine at the spot	7.08 ± 0.18	7.38 ± 0.43	6.26 ± 0.68	6.37 ± 0.76	0.28	0.001
Urinary NH_4_-N in jar without acid (g/kg)	7.89 ± 2.30	5.82 ± 4.83	6.60 ± 1.99	6.39 ± 2.37	0.38	0.77
Urinary total N in jar without acid (g/kg)	8.86 ± 2.59	6.92 ± 5.80	8.17 ± 1.94	8.14 ± 2.52	0.48	0.85
Urinary K in jar without acid (g/kg)	5.29 ± 2.07	5.24 ± 2.48	5.21 ± 0.47	5.87 ± 1.66	0.66	0.68
Urinary NH_4_-N in jar with acid (mg/kg)	849 ± 574	519 ± 320.68	648 ± 164	977 ± 364	0.09	0.63
pH of urine patches in jar with water at the spot	8.59 ± 0.50	8.25 ± 0.50	8.01 ± 0.01	8.14 ± 0.28	0.59	0.10
pH of urine soiled	8.46 ± 0.13	7.89 ±0.22	7.83± 0.44	7.76 ± 0.55	0.12	0.07
NH_4_-N of urine patches in jar with water (mg/kg)	3483 ± 3447	7338 ± 1121	3451 ± 1753	2879 ± 1964	0.51	0.40
K of urine patches in jar with water (mg/kg)	1152 ± 526	672 ± 347	697 ± 480	1209 ± 705	0.94	0.85
NH_4_-N of urine patches in jar with acid (mg/kg)	2737 ± 1538	1391 ± 1677	1940 ± 1182	1800 ± 1215	0.09	0.63
pH of upper manure in jar with water at the spot	7.90 ± 0.21	7.90 ± 0.20	8.00 ± 0.01	8.03 ± 0.05	0.12	0.77
NH_4_-N of upper manure in jar with water (mg/kg)	4151 ± 1235	2891 ± 1135	2548 ± 1328	3562 ± 1096	0.58	0.06

CON = control diet, RCP = reduced crude protein diet, AD = acidifying diet, CD = combined diet.

**Table 6:** Effects of experimental diets on manure characteristics (mean ± standard deviation, *n* = 4).

Items	CON	RCP	AD	CD	*p*-Value	
					Protein	Acidifying
pH at the spot	7.39 ± 0.46	6.94 ± 0.08	7.03 ± 0.12	6.99 ± 0.04	0.06	0.20
Dry matter (g/kg)	97.7 ± 9.2	93.2 ± 8.9	86.4 ± 15.9	104.5 ± 10.4	0.15	0.99
Ash (g/kg)	18.90 ± 1.56	19.27 ± 1.62	18.77 ± 2.40	19.65 ± 1.38	0.30	0.83
NH_4_-N (g/kg)	4.03 ± 0.51	3.23 ± 0.52	4.62 ± 0.73	3.90 ± 0.47	0.02	0.04
Total-N (g/kg)	6.88 ± 0.53	5.93 ± 0.58	7.28 ± 0.59	6.75 ± 0.42	0.04	0.07
K (g/kg)	3.98 ± 0.39	3.70 ± 0.10	4.19 ± 0.18	4.08 ± 0.45	0.20	0.07
Na (g/kg)	0.65 ± 0.09	0.64 ± 0.09	0.69 ± 0.02	0.68 ± 0.10	0.82	0.43
P (g/kg)	1.32 ± 0.12	1.24 ± 0.09	1.18 ± 0.15	1.36 ± 0.17	0.44	0.87
Cl (g/kg)	0.64 ± 0.07	0.69 ± 0.06	0.74 ± 0.08	0.67 ± 0.17	0.91	0.51
Ca (g/kg)	1.89 ± 0.14	2.25 ± 0.52	1.85 ± 0.65	2.25 ± 0.38	0.06	0.90
Acetic acid (g/kg)	6.56 ± 1.86	6.79 ± 1.91	7.18 ± 2.05	9.35 ± 0.90	0.19	0.09
Propionic acid (g/kg)	2.44 ± 0.37	2.97 ± 0.62	2.56 ± 0.61	2.62 ± 0.46	0.26	0.64
Iso-Butyric acid (g/kg)	0.72 ± 0.08	0.70 ± 0.10	0.72 ± 0.17	0.63 ± 0.08	0.28	0.46
Butyric acid (g/kg)	3.04 ± 0.67	2.65 ± 0.40	2.36 ± 0.89	3.24 ± 0.74	0.31	0.85
Iso-Pentanoic (g/kg)	1.00 ± 0.09	0.97 ± 0.11	1.00 ± 0.18	0.96 ± 0.10	0.41	0.82
Pentanoic acid (g/kg)	0.38 ± 0.07	0.49 ± 0.09	0.35 ± 0.12	0.41 ± 0.08	0.08	0.25
Total VFA (g/kg)	14.14 ± 2.76	14.57 ± 2.52	14.17 ± 3.70	17.20 ± 2.27	0.19	0.31
Buffer capacity	0.026 ± 0.003	0.040 ± 0.005	0.036± 0.004	0.034± 0.004	0.001	0.10
Manure TIC (mol/kg)	0.184 ± 0.034	0.114 ± 0.016	0.105 ± 0.008	0.112 ± 0.017	0.009	0.002

CON = control diet, RCP = reduced crude protein diet, AD = acidifying diet, CD = combined diet, VFA: volatile fatty acids, TIC: total inorganic carbon.

**Table 7:** pH and ammonia emissions from growing-finishing pig house as affected by experimental diets (mean ± standard deviation, *n* = 4).

Items	CON	RCP	AD	CD	*p*-Value	
					Protein	Acidifying
pH at 0.5 cm depth from the manure surface	8.48 ± 0.41	7.56 ± 0.13	7.79 ± 0.13	8.01 ± 0.52	0.07	0.49
pH at 5 cm depth from the manure surface	7.79 ± 0.31	6.94 ± 0.23	7.19 ± 0.08	7.11 ± 0.23	0.003	0.09
Ammonia concentration at 1 cm above ( the manure surface (mg/m^3^)	15.66 ± 6.12	8.02 ± 2.19	9.89 ± 1.82	6.50 ± 1.43	0.02	0.09
Ammonia concentration at 10 cm above (the manure surface (mg/m^3^)	9.55 ± 3.31	7.23 ± 2.53	8.96 ± 3.43	5.79 ± 1.83	0.02	0.42
NH_3_ emission from the floor (g/day)	1.91 ± 1.18	1.03 ± 0.67	1.34 ± 1.19	1.35 ± 0.90	0.06	0.41
NH_3_ emission from animal house (g/day)	3.90 ± 1.38	2.68 ± 1.01	3.03 ± 1.57	3.03 ± 0.74	0.08	0.42

CON = control diet, RCP = reduced crude protein diet, AD = acidifying diet, CD = combined diet.

## Data Availability

The data presented in this study are available on request from the corresponding author. The data are not publicly available due to data are still being processed to produce other papers.
