# Correction: Le Dinh et al. Effect of Diet Composition on Excreta Composition and Ammonia Emissions from Growing-Finishing Pigs. *Animals* 2022, *12*, 229

**DOI:** 10.3390/ani12070902

**Published:** 2022-04-01

**Authors:** Phung Le Dinh, Carola M. C. van der Peet-Schwering, Nico W. M. Ogink, André J. A. Aarnink

**Affiliations:** 1 Wageningen Livestock Research, Wageningen University and Research, 6708 WD Wageningen, The Netherlands; ldphung@hueuni.edu.vn (P.L.D.); gvdpeet@wxs.nl (C.M.C.v.d.P.-S.); nico.ogink@wur.nl (N.W.M.O.); 2 Faculty of Animal Sciences and Veterinary Medicine, University of Agriculture and Forestry, Hue University, 102 Phung Hung, Hue, Vietnam

**Error in Table**

In the original publication [1], there was a mistake in Table 1 as published. Rows were omitted or shifted. Therefore, a few values are not located in the right places in the table. The corrected Table 1 appears below.

The authors apologize for any inconvenience caused and state that the scientific conclusions are unaffected. The original publication has also been updated.

## Figures and Tables

**Table 1:** Ingredient composition of experimental diets, as-fed basis.

Composition (%)	Starter Diet				Grower Diet				Finisher Diet			
	CON	RCP	AD	CD	CON	RCP	AD	CD	CON	RCP	AD	CD
Maize	11.32	12.81	11.79	12.81	5.40	5.00	5.00	5.00	5.00	5.00	5.40	5.00
Barley	24.63	24.63	24.63	24.63	19.80	22.70	19.80	22.00	19.80	19.80	19.80	19.80
Wheat	17.50	16.75	14.78	16.75	27.45	26.80	25.07	24.80	27.65	29.64	24.56	28.74
Maize feed meal	5.00	4.81	5.00	4.41	4.50	4.95	4.75	4.95	4.50	4.95	4.50	4.95
Wheat middlings		3.39	1.01	4.21	7.00	9.90	7.00	9.90	7.00	10.00	7.00	10.00
Biscuits, ground	4.93	4.38	4.93	3.94	4.30	4.00	5.00	5.00	3.50	2.80	4.50	3.00
Wheat gluten feed meal	7.30	6.90	7.22	6.90	5.00	5.00	5.00	5.00	4.50	4.50	4.50	4.90
Vegetable oil	1.61	1.92	1.82	1.90	1.50	1.60	1.95	2.00	1.00	1.00	1.40	1.35
Potato protein		0.28										
Sugar beet pulp					1.10	1.30	1.10	1.30	1.10	1.50	1.10	1.20
Soybean meal HIPRO	17.29	14.87	17.77	16.04	10.10	7.90	11.00	8.30	8.50	5.00	9.00	5.00
Soybean hulls		1.04		0.77	1.30	2.00	1.30	2.00	1.00	1.00	1.00	1.00
Rapeseed meal, fat extract	4.93		4.93		5.00		5.00		4.80	1.00	5.00	1.10
Sunflower seed meal, fat extract	1.48	0.99	1.21		4.00	2.90	3.20	3.00	3.50	3.50	3.50	3.60
Palm kernel flakes									4.40	4.90	3.80	5.00
Whey, concentrated	0.40	3.00	0.10	2.07	1.10	3.00	1.10	2.80	1.70	3.00	1.70	1.70
Limestone	1.14	1.11	0.30	0.30	0.89	0.89			0.93	0.96	0.13	0.18
Salt	0.28	0.20	0.29	0.21	0.20	0.22	0.20	0.12	0.19	0.09	0.19	0.08
Vitamin extra					0.19	0.19	0.19	0.19				
Premix ^1^	0.25	0.25	0.25	0.25	0.26	0.26	0.26	0.26	0.25	0.25	0.25	0.25
DL-Methionine	0.09	0.17	0.10	0.21	0.03	0.08	0.03	0.08		0.03		
l-Lysine HCL	0.52	0.71	0.50	0.70	0.41	0.61	0.38	0.60	0.29	0.51	0.28	0.51
l-Threonine	0.14	0.22	0.14	0.22	0.09	0.17	0.09	0.17	0.04	0.12	0.04	0.12
l-Tryptophan	0.05	0.17	0.05	0.16		0.09		0.08				
Valine		0.29		0.30								
Benzoic acid			1.00	1.00			1.00	1.00			1.00	1.00
Calcium formate			1.04	1.03			1.00	1.00			1.00	1.00

CON = control diet, RDP = reduced crude protein diet, AD = acidifying diet, CD = combined diet = reduced crude protein + acidifying diet. ^1^ The vitamin–mineral premix supplied per kg feed included 7000 IU vitamin A, 1700 IU vitamin D3, 20 IU vitamin E, 1.5 mg vitamin K, 1.5 mg vitamin B1, 4 mg vitamin B2, 11 mg d-pantothenic acid, 18 mg niacine, 18 µg vitamin B12, 0.1 mg folium acid, 1.0 mg vitamin B6, 100 mg choline chloride, 75 mg Fe, 10 mg Cu, 65 mg Zn, 30 mg Mn, 0.15 mg Co, 0.75 mg I, 0.30 mg Se.
